# Multi-Scale Quality Evaluation of Road Markings from Glass Bead Distribution: A Novel GSMTA Framework

**DOI:** 10.3390/ma19112244

**Published:** 2026-05-26

**Authors:** Xiaosong Lu, Haoqin Guo, Rui He, Hao Wu, Liangliang Li, Jianrong Hu

**Affiliations:** 1 School of Materials Science and Engineering, Chang’an University, Xi’an 710061, China; 2021031007@chd.edu.cn (X.L.); 2024031006@chd.edu.cn (H.G.); 2021031015@chd.edu.cn (L.L.); 2 The Engineering Design Academy of Chang’an University Co., Ltd., Xi’an 710061, China; jhu87164@gmail.com

**Keywords:** road safety, road markings, distribution evaluation, segmentation, morphology, retroreflectivity

## Abstract

**Highlights:**

- YOLOv8-based instance segmentation improves the accuracy of GBs by 15% compared to traditional methods.
- Three-dimensional evaluation system links GB distribution to *R*_L_ (R^2^ = 0.93).
- The L3D index has been experimentally validated to predict *R*_L_, with a prediction error below 10%.

**Abstract:**

Road markings constitute essential traffic control elements that ensure safety and traffic flow efficiency. The nighttime visibility of road markings, quantified through retroreflective luminance (*R*_L_), is fundamentally governed by the distribution characteristics of embedded glass beads (GBs) within the marking matrix. Yet, three persistent limitations hinder reliable GB distribution evaluation: measurement variability, oversimplified model assumptions (fixed 50% embedment depth vs. observed 50–60% variations), and fragmented correlations between GB morphology and *R*_L_ metrics. This study proposes a granulometric–spatial–morphological triad assessment (GSMTA) framework, integrating instance segmentation with hierarchical performance analytics. The GSMTA framework achieves 15% higher segmentation accuracy over Otsu/Fast Random Forest methods, quantifying GB distribution via granulometric (size gradation), spatial (homogeneity index), and morphological (shape factor) descriptors. Through principal component analysis, the derived L^3^D performance indices establish statistically robust retroreflective luminance (*R*_L_) prediction models, with PC1 and PC2 capturing 91.7% of the variance and validation errors. The model remained below 8% error across a 750 mcd·m^−2^·lx^−1^ *R*_L_ range, ensuring reliable and precise performance evaluation. Field validation demonstrates the framework’s capacity of transforming pixel-level segmentation data into practical quality control metrics. This advancement supports lifecycle management through standardized GB distribution evaluation, overcoming prior incompatibility issues between microscopic morphology analysis and macroscale *R*_L_ measurements.

## 1. Introduction

Road markings are vital elements of traffic safety infrastructures, playing a pivotal role in lane delineation and visual guidance for road users, significantly enhancing traffic efficiency and safety. The retroreflective luminance (*R*_L_) of road markings, primarily enabled by embedded glass beads (GBs) in their surface, directly influences nighttime visibility and road user safety [1,2,3,4,5]. During the application of road markings, GBs are integrated either by pre-mixing into the matrix or through surface application via spreading devices prior to the solidification of the marking material. Surface-applied GBs undergo sedimentation under the dynamic equilibrium between gravitational force and the viscous resistance of the uncured substrate, ultimately forming a specific settling state. In ideal conditions, the GBs exhibit homogeneous surface dispersion and achieve a reference embedding rate of 50–60% within the road markings [6,7]. However, practical outcomes are modulated by multiple interdependent factors: ambient temperature and humidity alter the viscoelastic properties and curing rate of the marking matrix, while GBs’ specific attributes (granulometric properties, bulk density, and surface modification methods) govern interfacial adhesion and settling behavior [8]. Additional variability stems from the construction personnel and the mechanical precision of equipment, collectively influencing the GB distribution characteristics [9]. Consequently, evaluating construction quality through isolated assessment of environmental conditions, material properties, or operational parameters introduces inherent limitations. As GB distribution serves as the ultimate manifestation of these interdependent factors, quantitative analysis of its distribution has emerged as a critical evaluation for systematically assessing both construction and service quality in road markings.

As the relationship between GB distribution characteristics and *R*_L_ has been widely acknowledged, existing approaches for GB assessment and *R*_L_ prediction typically employ imaging techniques (e.g., light detection and ranging, 905 nm or 1550 nm LiDAR and MATLAB-based segmentation [10]) or data-driven degradation models [11,12,13,14,15]. These methods face three critical limitations that constrain their operational validity. Firstly, uncontrolled variability in field measurements and human visual inspections exhibit inter-operator disagreement in GB distribution assessment, while LiDAR-based techniques, despite their technological sophistication, demonstrate unstable error margins (the standard deviation is more than 30 times higher than the average error). Secondly, oversimplified assumptions in analytical models lead to systematic prediction biases. For instance, a computer-vision-based visibility estimation framework relied on a fixed 50% bead embedment depth assumption, a parameter shown to vary between 50~60% depending on application techniques, thereby inducing empirical deviations in *R*_L_ estimations [10]. Thirdly, the lack of multi-scale performance unification creates fragmented interpretations: no standardized framework exists to reconcile microscale GB morphology (via camera), spatial distribution, and macroscale granulometric properties with *R*_L_, resulting in incomparable studies that hinder systematic evaluation of road marking based on GBs. In addition, Babić et al. emphasized that systematic evaluation frameworks are critical for enhancing the sustainability and lifecycle performance of road marking systems based on a longitudinal study spanning over two decades [16,17,18,19]. The critical role of systematic distribution evaluation of GBs in ensuring construction efficacy and service reliability has been underestimated [20,21,22,23,24].

Addressing these limitations necessitates a strategy integrating precise segmentation of GBs, determination of model parameters, and multi-scale evaluation. Nevertheless, measuring the distribution of GBs in road markings faces a series of challenges due to the small size, dense distribution and complex reflection, refraction, and retroreflection phenomena [25,26,27,28]. As deep-learning-based image processing technologies are now extensively applied across transportation, remote sensing, and autonomous driving [29,30], all processes that require segmentation of objects, instance segmentation models have shown the potential to achieve pixel-level segmentation of densely distributed GBs. Thus, this study selects the YOLOv8 framework as the foundational architecture for the instance segmentation task due to its fast, accurate, one-stage, and anchor-free characteristics [31,32,33,34]. Then, the research team annotated datasets of road marking GBs, and trained instance segmentation models. Then, the masks of GBs were generated via the trained models. The granulometric, spatial, and morphological properties of GBs were obtained from the segmented masks and utilized as the major parameters to comprehensively reflect the distribution characteristics of GBs. Building upon the need for systematic evaluation methodologies, this study establishes a granulometric–spatial–morphological triad assessment (GSMTA) framework for GB distribution in road markings by integrating YOLOv8-driven segmentation with multi-scale analysis.

The GSMTA framework specifically targets the limitations through three components: (1) training a pixel-level segmentation model and comparative validation, (2) multi-scale parameter analysis of GBs, and (3) the development of a hierarchical evaluation system integrating multi-scale parameters. This GSMTA framework enables the transformation of raw segmentation data into quantifiable metrics predictive of retroreflective performance, providing actionable insights for quality control in road marking applications.

## 2. Materials and Methods

### 2.1. Materials

#### 2.1.1. Properties of Raw Materials

Methyl Methacrylate (MMA)-based two-component coatings and GBs with a refractive index (RI) of 1.7 were purchased from Anhui Tory Materials Technology Inc. (Bengbu city, China), and utilized to prepare road markings. The specifications for these GBs are provided in Table 1.

#### 2.1.2. Gradation Design of GBs

The experiment on the distribution of GBs involved the use of both individually sieved single-size and mixed-gradation GBs. The particle sizes of single-size GBs had ranges of 1250~1000 μm, 1000~800 μm, 800~600 μm, and 600~300 μm. Subsequently, the GBs of these four gradations were blended in proportions to obtain mixed gradation, as specified in Table 2. The mixing programs in Table 2 were designed by considering the collaborative functions of beads across different service stages to evaluate the influence of GB gradation on the distribution state and *R*_L_ of road markings. Larger beads are intended to maintain retroreflectivity under wet conditions and prevent excessive sinking during application, which helps ensure adequate initial retroreflection. Smaller beads contribute to sustaining a high retroreflection coefficient and work in conjunction with larger beads to prevent a sudden decline in performance as the marking undergoes wear. Additionally, narrower gradation ranges were designed to analyze the specific impact of the gradation span on the *R*_L_ coefficient, while the Mix-L and Mix-S programs were included to study the respective effects of relatively coarse and fine distributions on marking performance.

#### 2.1.3. Training Dataset Preparation

Photos of GBs were captured using a mirrorless camera with a 20-megapixel (20 MP) resolution, producing images with dimensions of 5569 × 3712 pixels. The shooting parameters were a 35 mm focal length, ISO 100, aperture of f/5.3, and a shutter speed of 1/60 s, utilizing a flash in return-light-detected mode. Additionally, a calibration block measuring 5 mm in length was photographed to annotate the actual dimensions of the images. The entire image, measuring 5569 × 3712 pixels, was cropped to extract a central area of 3200 × 1920 pixels, which was then cropped into fifteen 640 × 640-pixel segments arranged in a 5 × 3 grid. Cropped images were annotated using the LabelMe (5.4.1) [35] tool within a Python environment, and all Python-related work in this study was conducted using Python 3.8.18. Objects within the images were classified into two categories: GBs and background. For the instance segmentation dataset, only the GBs were required to be labeled, with the remaining areas automatically designated as background. The annotated files were saved in JSON format. These labeled JSON files were subsequently converted into YOLO-format datasets, comprising training and validation sets with a split ratio of 0.8:0.2. Following these steps, a micro-dataset containing 30 annotated images, a small dataset comprising 90 photographs, and a medium dataset with 150 annotated images were created. To prevent data leakage, the dataset partition was executed at the macro-image level. All sub-images (crops) originating from a single high-resolution photograph were strictly confined to the same subset (training and validation).

### 2.2. Research Method

#### 2.2.1. YOLOv8-Based Architecture

In this study, YOLOv8-seg was selected as the pixel-level feature extraction engine due to its anchor-free detection head and Decoupled Head architecture, which are particularly effective for detecting and segmenting densely packed small objects like glass beads. To optimize the model for the unique micro-textures of road markings, a hyperparameter evolution strategy was implemented. Key parameters, including the initial learning rate (lr0 etc.) and weight decay, were optimized using a genetic algorithm over 30 iterations.

#### 2.2.2. Performance Evaluation Indicators

To evaluate the YOLOv8-seg model’s efficacy in extracting glass bead features, Precision, Recall, Intersection-over-Union (IoU), and mean Average Precision (mAP) were utilized, each explicitly mapped to the challenges of marking inspection.

Precision and Recall assess the model’s fundamental recognition reliability against complex backgrounds. In this specific task, low Precision (driven by False Positives) implies that the model incorrectly identifies resin, exposed fillers, or localized light reflections as glass beads, which would inflate the calculated bead density. Conversely, Recall measures the model’s sensitivity to challenging targets. A low Recall (driven by False Negatives) indicates a failure to detect beads that are embedded in the binder or obscured by surface pollution, leading to an underestimation of the retroreflective potential [36,37].

Furthermore, precise morphological extraction relies heavily on pixel-level boundary accuracy, quantified by the Intersection-over-Union (IoU). The IoU measures the spatial overlap between the predicted mask and the ground truth. In our methodological framework, a high IoU is necessary as minor boundary deviations can severely distort the calculation of the shape factor and gradation of GBs.

Consequently, the overall segmentation capability is evaluated using mAP_50_ and mAP_50–95_. While mAP_50_ provides a baseline for GB localization, mAP_50–95_ (which averages the AP across IoU thresholds from 0.5 to 0.95) is also critical for this study. Because glass beads on road markings are often densely packed and physically touching, a high mAP_50–95_ guarantees the model’s capability to delineate adjacent instances. This boundary separation is the methodological prerequisite for accurately calculating the distribution characteristics, preventing GBs from being erroneously merged into a single massive entity. The F1 score was additionally reported to harmonize Precision and Recall, ensuring balanced feature extraction without extreme biases.

#### 2.2.3. Model Training and Hyperparameter Tuning and Training

The image size of the input dataset is 640 × 640 pixels. Throughout the training phase, the mosaic technique was utilized for data augmentation, entailing the random scaling, cropping, and arrangement of samples from the training dataset for combination. To enhance the detection and segmentation performance for dense, small-scale targets such as glass beads, a systematic hyperparameter optimization process was executed. Rather than relying on manual adjustment or a traditional grid search, this study utilized the genetic algorithm (GA)-based evolution functionality provided by the Ultralytics (8.0.197) framework (via the model.tune() method).

The optimization process involved 30 evolutionary iterations, where each generation performed a truncated training cycle to evaluate a specific hyperparameter configuration. The search space encompassed critical parameters, including the initial learning rate (η), momentum (μ), weight decay (λ), and data augmentation gains (e.g., mosaic and scaling factors). A composite fitness function, primarily weighted by mAP_50_ and mAP_50–95_, was employed to guide the selection of the fittest parameter set. This refinement ensured that the final model configuration was specifically tailored to the unique micro-morphological features and complex illumination conditions of road marking imagery, improving the model’s robustness and convergence efficiency.

#### 2.2.4. Spray Test for GBs

**Figure 1:**
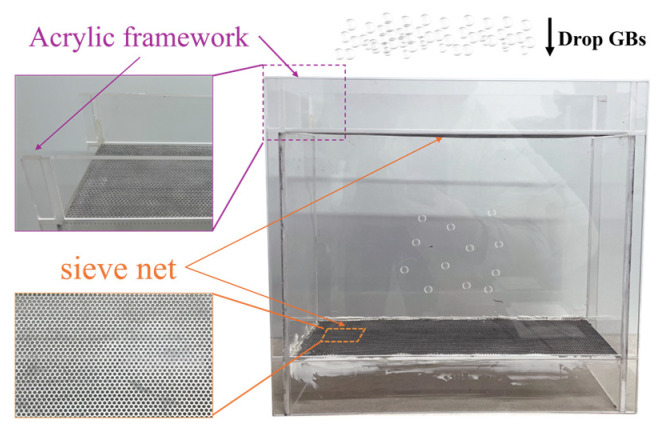
Structure of GB sprayer.

This study designed a bead-spreading device to mitigate the randomness and uneven distribution of GBs applied manually to traffic markings, as illustrated in Figure 1. The application settings for bead distribution were specified as follows: the distance from the lower screen to the surface of the coating was 20 cm, with a bead application rate of 500 g/m^2^. The timing for bead application was set to 5 s after the coating application, with the entire process of bead spreading completed within 10 s. The experiment was conducted at 20 °C, using an MMA-based two-component resin hardener at 2% of the coating mass. The thickness of the coating film is 0.6 mm. GBs with single-gradation groups (SD6182, SD6812, SD6682, SD6362) and mixed-gradation groups, identified as Mix-L1, Mix-L2, Mix-S1, and Mix-S2, were applied separately. The codes of the test groups are shown in Table 2. For example, SD6182 denotes that single-gradation (SD) GBs are applied on a 0.6 mm (6) coating film with particle sizes of 1180–1000 μm (18), and the distance between the lower screen and the film is 20 cm (2).

#### 2.2.5. Measurement of R_L_ of Coatings

The LTL-X MarkII (DELTA, Copenhagen, Denmark) road marking retroreflectometer was used to measure the *R*_L_ of the prepared experimental road markings and the field tests, and the average value was measured three times at each point. The irradiation angle of the *R*_L_ measured by the device is 1.24°, and the observation angle is 2.29°.

#### 2.2.6. Particle Analysis of GBs

This work employed FIJI (ImageJ 1.54f) for conventional image processing and machine-learning-assisted segmentation, utilizing both the Otsu method and the Fast Random Forest machine learning algorithm based on the Weka platform. These methods were then compared to the results obtained from YOLOv8 instance segmentation.

Particle analysis on the binary images obtained from segmentation involved using the mean of the major and minor axes of the fitted ellipse to determine particle diameter. Particles at the periphery or with a roundness below 0.2 were excluded to mitigate the distortion in particle shape due to image cropping.

#### 2.2.7. Evaluation of Distribution Uniformity

The assessment of distribution uniformity is divided into three levels. The first level pertains to the granulometric distribution of GBs. The second level focuses on the pixel’s spatial distribution characteristics of GBs, including the global uniformity of pixels and the distribution trend of GB particles. The third level concerns the morphological parameters of each GB. For Level 1, a gradation index (*GI*) is introduced to extract the distribution characteristics of single- and mixed-gradation GBs to showcase the size and distribution range of glass bead diameters. The *GI* is defined as the multiplication of the cumulative distributions within the intervals immediately before and after the midpoint of the distribution range, which corresponds to the peak of the GB diameter probability distribution curve. The definition of the *GI* is provided as follows:

(1) $$GI=g_{m}\times p_{c},$$

where *g_m_* represents the x-coordinate of the midpoint of the interval corresponding to the peak of the probability density curve, which is the point of the highest concentration of GB diameters. *p_c_* denotes the cumulative probability, encompassing the cumulative distribution across the intervals immediately preceding and following the *g_m_* point.

At Level 2, the assessment relies on the standard deviation of the total pixels (TPs) in the Region of Interest (ROI) for each segmented block throughout the entire image. After image preprocessing, segmentation, and masking, the total area of white pixels for each of the 15 image blocks is determined, as well as the average area across all blocks. The standard deviation of these white pixel areas is calculated and designated as the distribution homogeneity (*DH*) [38]. The definition of the *DH* is provided as follows:

(2) $$D_{H}=\sqrt{\frac{\sum_{i=1}^{n}{\left(TP_{i}-\bar{\mathrm{TP}}\right)}^{2}}{n-1}}/\frac{\sum_{i=1}^{n}TP_{i}}{n},$$

where *TP_i_* is the total number of white pixels in the *i*-th block, $\bar{\mathrm{TP}}$ is the mean of white pixels across all blocks, and *n* is the total number of blocks, with a value of 15 in this context.

For Level 2, unsupervised clustering methods are employed to analyze the distribution of GBs, as clustering analysis can assess the degree of particle agglomeration. For each image block, the number of clusters *n* is set to 2, 3, and 4, respectively, and clustering evaluation metrics are used to assess the results. A lower number of clustering (NC) outcomes indicates a higher degree of glass bead aggregation and uneven distribution. The MiniBatchKMeans method from the “sklearn” library is utilized for clustering, with “k-means++” initialization to enhance computational efficiency and achieve improved clustering outcomes [39].

The Calinski–Harabasz Index (CHI), also known as the Variance Ratio Criterion, evaluates the quality of clustering [40]. A higher CHI value indicates better clustering performance. The Davies–Bouldin Index is a metric that evaluates the average ‘similarity’ among clusters, with ‘similarity’ being the ratio of intra-cluster distances to inter-cluster distances [41]. A lower Davies–Bouldin Index indicates better clustering, as zero is the lowest possible score and signifies optimal clustering.

Individual particle separation is essential for particle morphological analysis at level 3. This is achieved by sequentially scanning each row of the binary image and examining every pixel within. Upon encountering a pixel with a value of 255, the boundary of the object comprising that pixel is outlined, and the object’s dimensions are measured, followed by the generation of a mask. This procedure is repeated in a loop. Utilizing the “regionprops” function from the scikit-image [42] library facilitates particle analysis, offering metrics such as each instance’s area, centroid coordinates, eccentricity, and perimeter. The shape factor (SHPT) for GBs, can be calculated using the following formula:

(3) $$SPHT=\frac{4\cdot \pi \cdot A}{p^{2}},$$

where *A* represents the area of GBs, and *p* is the perimeter of GBs.

### 2.3. Experimental Engineering Project

**Figure 2:**
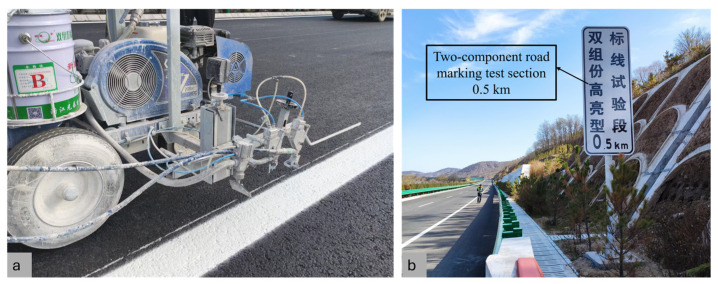
The test section of road markings in Yan’an city, where (**a**) is the construction process and (**b**) is the panorama of the test section.

The experimental engineering project was carried out on three cities, including Yan’an city (Shaanxi Province, China), Nanchang city (Jiangxi Province, China), and Taiyuan city (Shaanxi Province, China). MMA-based two-component coatings were used in Yan’an city to construct the test section, while thermoplastic coatings were used in Nanchang and Taiyuan city to construct comparison sections. As is shown in Figure 2, each of the test sections was 0.5 km long. The RIs of GBs used in Yan’an and Nanchang city is 1.7, and the RI of GBs used in Taiyuan city is 1.5. Each region was randomly sampled to measure the *R*_L_ of 50 points, and the corresponding 50 photos were taken for the measurement area.

## 3. Results and Discussion

### 3.1. Model Comparison

Table 3 presents the optimal hyperparameters determined through the model.tune() method. To specifically address the challenges associated with a limited dataset size and to mitigate the risk of overfitting, targeted regularization and augmentation strategies were strictly parameterized. The AdamW optimizer was selected to ensure stable gradient descent, with the initial learning rate (lr0) and momentum dictating the step size and directional stability of weight updates. Crucially, a specific weight decay was applied as L2 regularization to penalize overly complex network weights, forcing the model to learn the generalized physical features of glass beads rather than memorizing sample-specific noise. Furthermore, data augmentation techniques—namely mosaic (contextual background expansion), scale (size invariance), and fliplr (spatial diversity)—were utilized to expand the geometric variance of the training distribution. Finally, while the maximum epoch was set to 600, an early stopping mechanism (patience = 50) was enforced to automatically terminate training once validation performance plateaued, preventing empirical overfitting.

**Figure 3:**
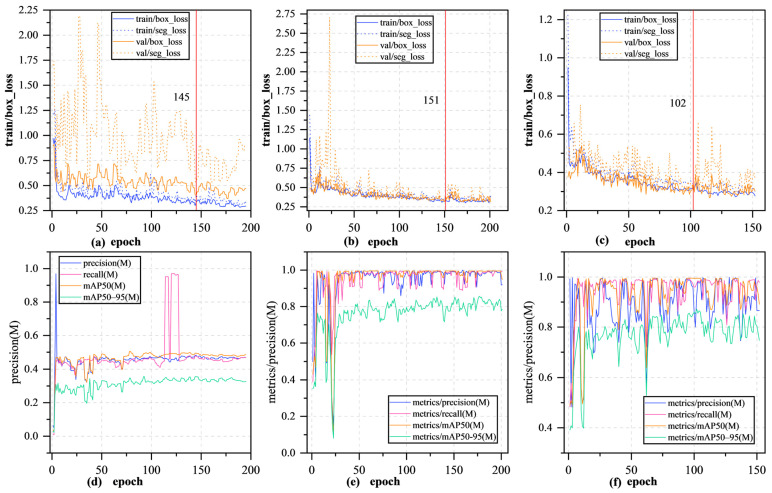
Training loss and validation loss of (**a**) 30 labeled images, (**b**) 90 labeled images, and (**c**) 150 labeled images; Precision, Recall, and mAP_50_ and mAP_50–95_ of (**d**) 30 labeled images, (**e**) 90 labeled images, and (**f**) 150 labeled images about masks.

The training dynamics and convergence behaviors of the YOLOv8-seg model were evaluated using three dataset scales (comprising 30, 90, and 150 labeled macro-images). To mitigate the risk of overfitting, an early stopping mechanism was implemented with a patience threshold of 50 epochs. Figure 3 illustrates the progression of bounding box and segmentation mask losses and the corresponding evaluation metrics (Figure 3a–c), as well as the Precision, Recall, and mAP_50_ and mAP_50–95_ of masks (Figure 3d–f) during the training process. The maximum training epoch was set to 600, but the training algorithms automatically terminated when validation metrics ceased to improve. Consequently, the optimal model weights were extracted at Epoch 145, Epoch 151, and Epoch 102 for the 30-, 90-, and 150-image datasets, respectively. The specific performance metrics at these optimal nodes are detailed in Table 4.

Analysis of the results reveals a clear correlation between the dataset volume and the model’s segmentation accuracy. For the constrained dataset of 30 images, the optimal model achieved an mAP_50_ (mask) of 0.4943. This baseline performance reflects the inherent difficulty of learning the complex micro-morphological features of glass beads when the training sample size is restricted and strict image-level data isolation is maintained.

As the dataset size increased, a substantial improvement in both segmentation accuracy and training stability was observed. The model trained on the 150-image dataset demonstrated the most favorable convergence efficiency, reaching its optimal state relatively early at Epoch 102. At this node, the model achieved an mAP_50_ (mask) of 0.9946 and a more stringent mAP_50–95_ of 0.8710. Concurrently, the validation bounding box loss and segmentation loss stabilized at lower levels (0.3391 and 0.3411, respectively) compared to those of the smaller datasets (e.g., 0.4046 and 0.5367 for the 30-image dataset). These metrics indicate that the expanded dataset, in conjunction with hyperparameter optimization (such as L2 regularization) and the early stopping protocol, effectively constrained empirical overfitting. The findings suggest that the finalized model possesses a sufficient level of robustness and accuracy for segmenting dense glass bead distributions under the evaluated experimental conditions.

**Figure 4:**
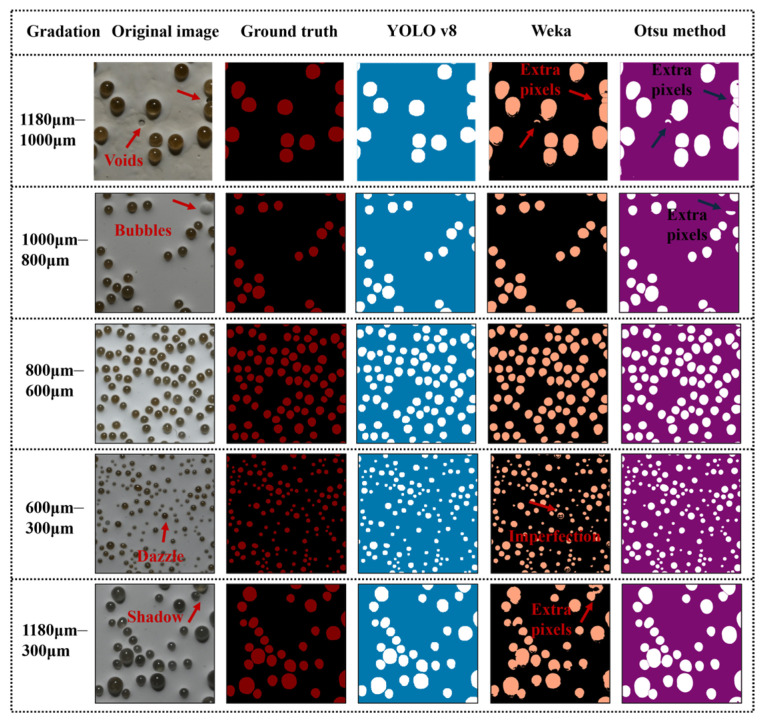
Comparison of the results using YOLOv8, Weka, and Otsu methods for GB segmentation.

The newly collected images of GBs were compared against manually annotated masks of GBs, the segmentation results from YOLOv8, the Fast Random Forest algorithm based on the Weka platform, and the Otsu method, as illustrated in Figure 4. A horizontal comparison of five images within the 1180–1000 μm range reveals that the segmentation results of the YOLOv8-based model most closely resemble the true values, successfully learning the characteristics of GBs and ignoring holes in the central marking paint and the top right corner. Conversely, both the Weka and Otsu methods misidentified holes as GBs, and particles in the top right corner were merged into a larger particle, which was then segmented using the watershed algorithm. Moreover, shadows cast by the GBs, obstructing light, were mistakenly identified as GBs by both the Weka and Otsu methods, leading to an overestimation in both the count and area of GBs by these segmentation approaches.

In the range of 1000–800 μm, the segmentation results of the three methods are similar where shadows are minimal. However, the Otsu method failed to exclude bubbles generated in the top right corner of the coating, erroneously identifying them as part of the GBs. For the uniformly sized, shadow-free 800–600 μm group, all three segmentation methods achieved good results. However, the Weka segmentation model showed incomplete segmentation with adhesion masks of GBs in the significantly varied 600–300 μm group. As is shown in Figure 4, Otsu segmentation included excessive pixels, inaccurately reflecting the gradation of GBs.

**Figure 5:**
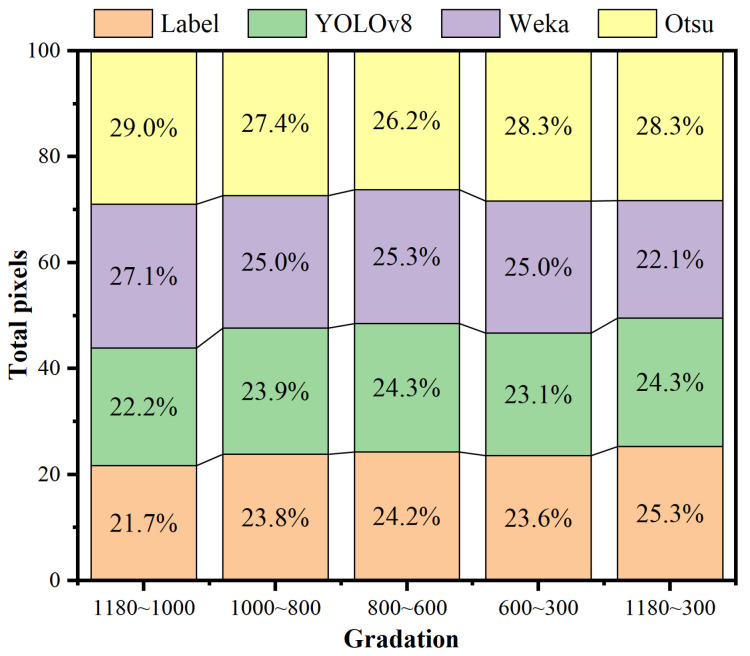
The total number of pixels obtained by different segmentation methods.

The total number of pixels resulting from different segmentation methods is shown in Figure 5. Overall, the segmentation results from YOLOv8 most precisely align with the annotated labels (ground truth). The Otsu and Weka methods both demonstrate a notable surplus of pixels, with a 1–5% error for the Weka method and 2–7% error for the Otsu method; the segmentation performance of different segmentation methods is shown in Table 5.

### 3.2. Granulometric–Spatial–Morphological Triad Distribution Analysis

To comprehensively assess the distribution state of GBs applied to road markings, granulometric properties, spatial distribution characteristics, and morphological factors are selected as key analytical metrics. These parameters are employed to evaluate the properties of the segmented GB image masks across three distinct hierarchical levels.

#### 3.2.1. Level 1—Granulometric Distribution Analysis

**Figure 6:**
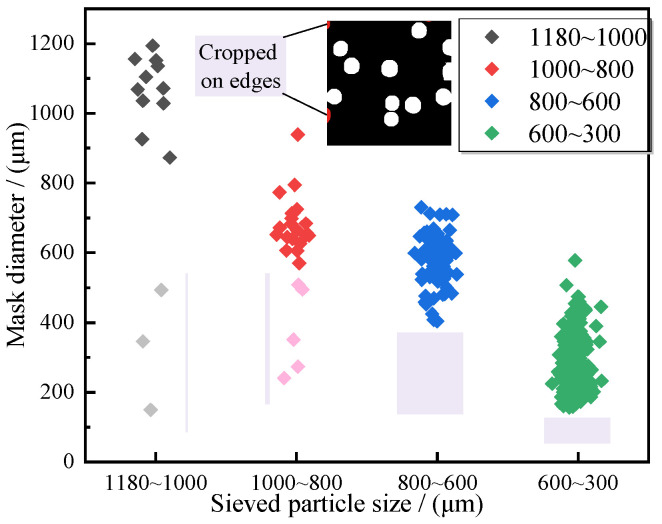
The particle distribution state of YOLOv8 segmentation.

The equivalent diameter of particles, and the distribution of particle size is illustrated in Figure 6. The alignment between segmented images and actual values is accurate within narrow particle size ranges. The particle size distribution derived from segmentation features some outliers beyond the expected range of GBs. This variation arises from GBs being embedded in the road markings, revealing only a fraction of their diameter, which is less than the predefined gradation range, or from some beads being partially truncated during the image cropping process. These instances are depicted as particles situated within the gray columns in Figure 6.

**Figure 7:**
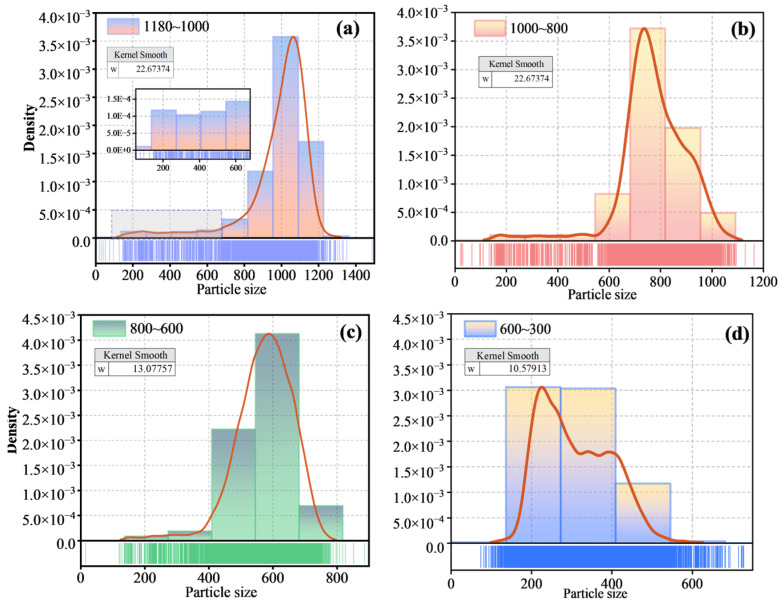
The distribution probability of single-gradation GBs, (**a**) 1180~1000mm, (**b**) 1000~800mm, (**c**) 800~600mm, (**d**) 600~300mm.

The distribution probability of single-gradation GBs is depicted in Figure 7. For single-gradation GBs, the distribution probability is concentrated near the lower limit of the particle size range. For GBs in the range of 1180–1000 μm, beads with a midpoint of the distribution diameter at 1023 μm account for 72.15%.

**Figure 8:**
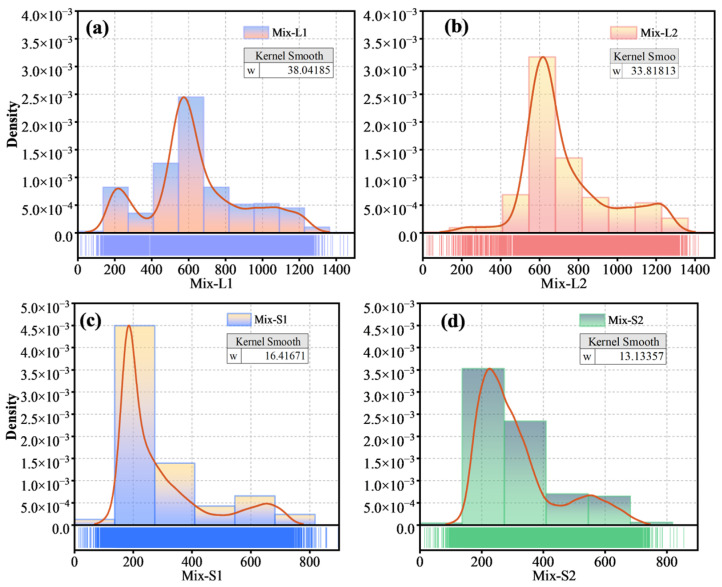
The distribution probability of mixed-gradation GBs, (**a**) Mix-L1, (**b**) Mix-L2, (**c**) Mix-S1, (**d**) Mix-S2.

The distribution probability for mixed-gradation GBs is shown in Figure 8. It is evident from Figure 8 that the particle size distribution range of mixed-gradation GBs is wider. Specifically, for Mix-S1 and Mix-S2, the midpoint of the particle size distribution range is positioned at 204 μm, whereas for Mix-L1 and Mix-L2, the midpoint is located at 614 μm.

According to the definition of the *GI*, it reflects the concentration of particle size distribution. The *GI* derived from image segmentation is presented in Table 6 for both single-gradation and mixed-gradation GBs.

#### 3.2.2. Level 2—Spatial Distribution Analysis

**Figure 9:**
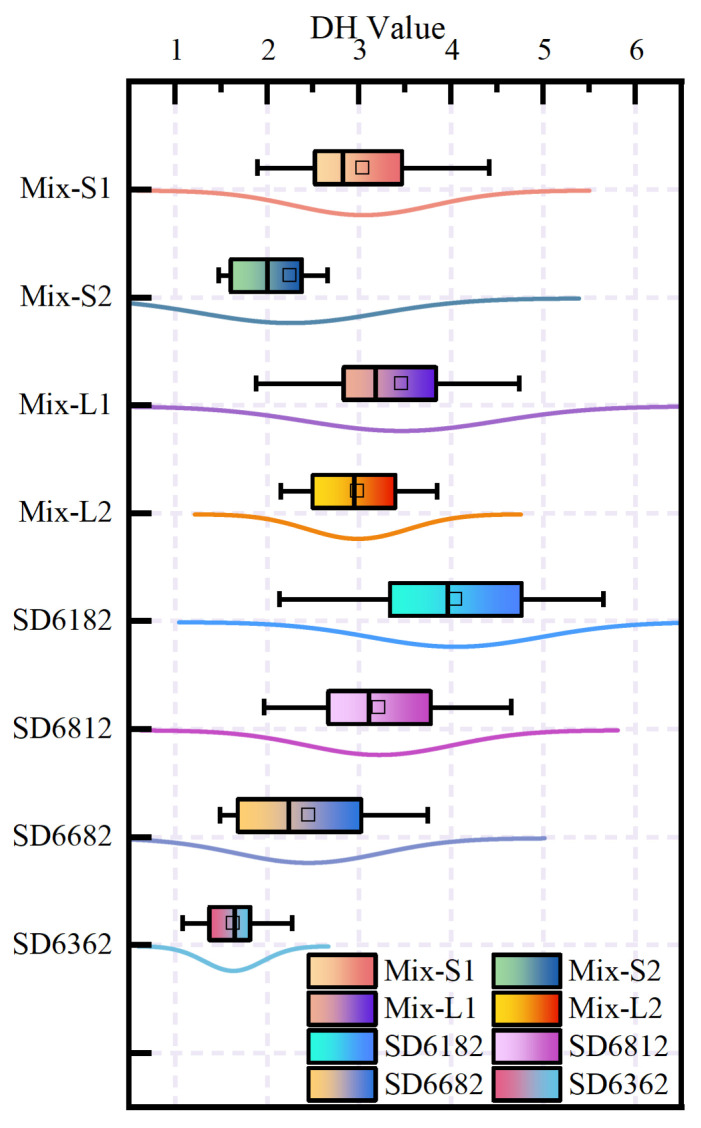
Distribution homogeneity (DH) of drop on GBs. The vertical line within each box represents the median, while the edges of the box indicate the 25th (Q1) and 75th (Q3) percentiles.

The evaluation of a pixel’s spatial distribution encompasses assessments of overall distribution homogeneity (DH) and pixel aggregation characteristics (NC). The initial step involves assessing the dispersion of pixels within the masks of segmented images. Each image is divided into 15 sections, and the uniformity of pixel distribution is calculated using the DH formula. The distribution trends visible in Figure 9 indicate that the distribution uniformity for larger particle sizes is lower than that for smaller gradations.

For Mix-S1 and Mix-S2, as illustrated by the gradation data, the initial gradation of the former is more uniform. However, Mix-S2 exhibits a lower DH value, indicating a more uniform distribution of image pixels. This may be attributed to Mix-S1 having a greater quantity of particles in the 100–300 μm range than Mix-S2, which has more particles in the 300–600 μm range, accounting for 60%. The exceedingly fine particles of 100–300 μm GBs, sinking almost entirely into the coating during dispersion, alter the rheological properties of the coating’s upper layer, thus modifying the embedding degree of beads in the 300–600 μm and 600–800 μm ranges. A larger quantity of finer 100–300 μm GBs leads to decreased distribution uniformity, i.e., a higher DH value. Regarding Mix-L1 and Mix-L2, the former has a more uniform initial gradation. Still, the latter exhibits a smaller DH after embedding, indicating a more uniform distribution from the perspective of pixels in the collected images. This variation stems from the coarser particle gradation of the former, characterized by a lower particle count than the latter. Moreover, based on the distribution characteristics of single-gradation particles, larger particles tend to form a more uneven distribution on the coating. In summary, the DH reflects the impact of glass bead particle size, quantity, and gradation characteristics on distribution uniformity.

**Figure 10:**
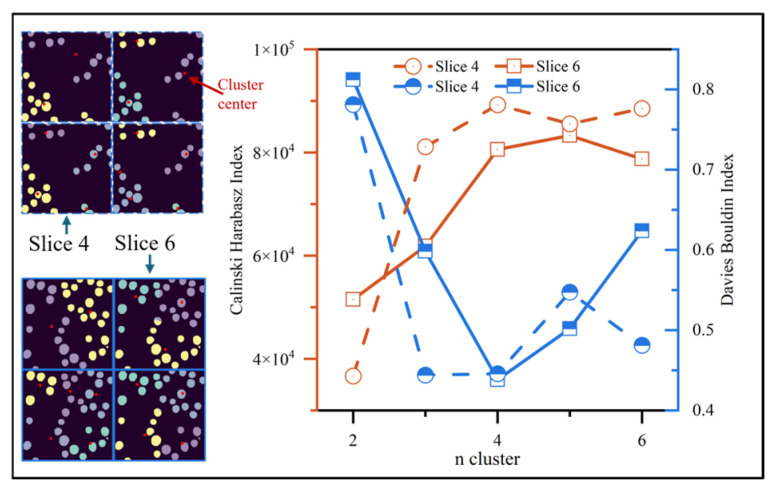
Clustering of cropped image slices, i.e., Slice 4 and Slice 6 in SD6812.

**Figure 11:**
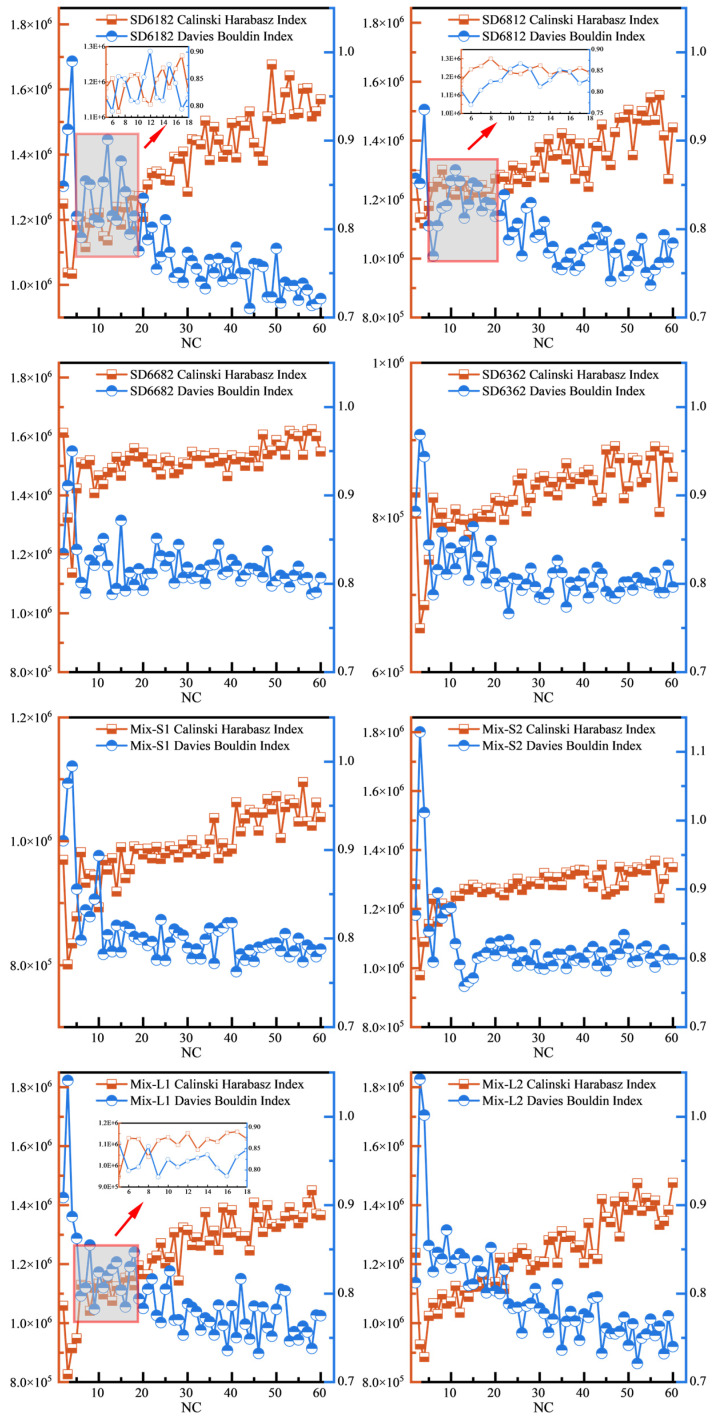
CHI and DBI of mixed-gradation and single-gradation GBs with different NCs.

Subsequently, clustering algorithms were utilized to evaluate the aggregation state of GBs. K-means clustering was applied to the slices obtained from the images, with the NC, ranging from two to six. The analysis based on the CHI and DBI is presented in Figure 10. According to the definitions of the CHI and DBI, a larger value of the CHI and a smaller value of the DBI indicate better clustering effectiveness. The results demonstrate that the optimal clustering performance for both slices was achieved with four clusters. Given the significant computational demands of clustering algorithms on pixel data from images, with slices consisting of several hundred thousand pixels (640 × 640) and the entire images comprising several million pixels (3200 × 1920), the NC for the whole image was varied from 2 to 60 (15 slices × 4), with the optimal number of clusters selected based on CHI and DBI values. The clustering analysis results for both single-gradation and mixed-gradation binary images are illustrated in Figure 11.

As the number of clusters increases, both the CHI and DBI exhibit fluctuating trends. The CHI and DBI were normalized to the [0, 1] range and then subtracted from each other to determine the optimal NC. The peak of the resulting curve indicates the optimal NC, and the optimal NC for each experimental group is presented in Table 7.

Upon analyzing the data distribution, Mix-S2 shows the minimal optimal cluster count, succeeded by SD6362, aligning with their reduced DH values and GI. SD6182, characterized by the largest average particle size, does not possess the maximum cluster quantity, presumably due to having the lowest number of particles, leading to a decreased optimal cluster count. Groups with coarser particle gradations exhibit elevated CHI peaks, and their DBI values tend to be 0, suggesting superior clustering efficiency in these cases.

#### 3.2.3. Level 3—Morphological Analysis of GBs

**Figure 12:**
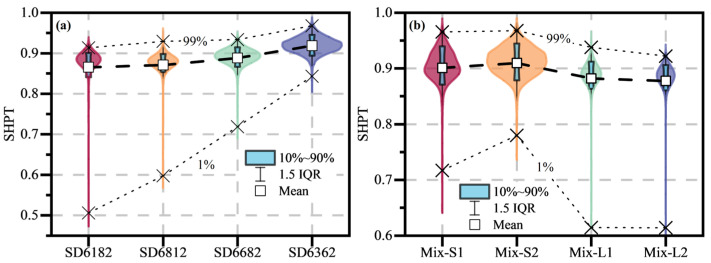
SHPT of experimental groups with single (**a**) and mixed (**b**) gradation.

At the third level, particle analysis focuses primarily on the morphology (shape factor) of glass beads. The “regionprops” method from the scikit-image library is employed to obtain the perimeter and area parameters for each glass bead in segmented masks, from which the SHPT values are calculated, as illustrated in Figure 12. The violin plots show the distribution of SHPT values and the corresponding quantity of glass beads for each experimental group.

It is observed that there is a deviation between the median point and the point of maximum probability density of the SHPT, which is more pronounced in experimental groups with coarser gradations. This indicates that GBs with larger average diameters have an increased probability of exhibiting lower SHPT values after embedding, reducing the mean value of the SHPT. This phenomenon can be attributed to two main factors: firstly, larger GBs are more challenging to form into spherical particles during the manufacturing process [43], and secondly, larger GBs are more likely to create shadowed areas in the captured images, resulting in obscure boundaries in the segmentation process, adversely affecting the SHPT.

### 3.3. L^3^D Indicator of GSMTA

Based on the three-level hierarchical evaluation of GB distribution in Section 3.2, multi-level assessment indicators, X={GI, DH, NC, SHPT}, have been established to characterize the distribution of glass beads from different geometric dimensions. Principal component analysis (PCA) was utilized to extract independent comprehensive variables to address potential multicollinearity among these variables and simplify the subsequent modeling process while retaining the primary information of the original dataset.

**Figure 13:**
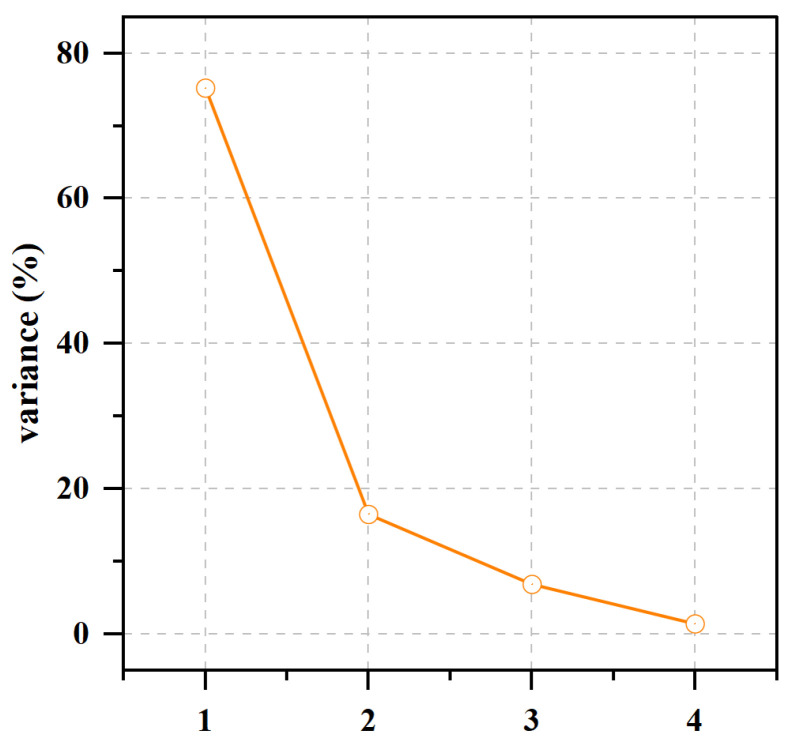
Scree plot of eigenvalue’s variance proportion.

Prior to the PCA calculation, the dataset **X** was normalized and centered to eliminate dimensional scales. The eigenvalues of the covariance matrix *Cov*(**X**) are presented in Table 8, and a scree plot (Figure 13) is introduced to visualize the eigenvalue trajectory. As indicated in Table 8, the first principal component (PC1) accounts for 75.21% of the total variance, and the second principal component (PC2) accounts for 16.49%. The cumulative variance contribution rate of the first two components reaches 91.70%, suggesting that they encompass the majority of the variance from the original indicators. Furthermore, the scree plot exhibits a distinct inflection point (elbow) after the second component, with the eigenvalues of the third and fourth components decreasing to marginal levels. Based on these statistical criteria, PC1 and PC2 were selected as the target dimensions for the subsequent modeling.

Beyond mathematical dimensionality reduction, these principal components can be associated with the physical state of the road marking system. Within the eigenvector of PC1, the coefficients corresponding to the GI, DH, and NC are positive, indicating a positive correlation with *R*_L_. In contrast, the SHPT exhibits a negative loading. In the context of 2D image segmentation for dispersed beads, a higher measured sphericity (SHPT) typically corresponds to glass beads that are deeply embedded within the coating or possess relatively small, exposed cross-sections. Both physical conditions restrict the effective optical area available for retroreflection [44]. Consequently, PC1 primarily represents the overall macro-spatial reflective capability of the GB system, where a higher PC1 score indicates a more favorable spatial distribution and embedment depth. PC2, accounting for the remaining variance, primarily reflects the secondary micro-morphological variations, such as localized structural arrangements.

**Figure 14:**
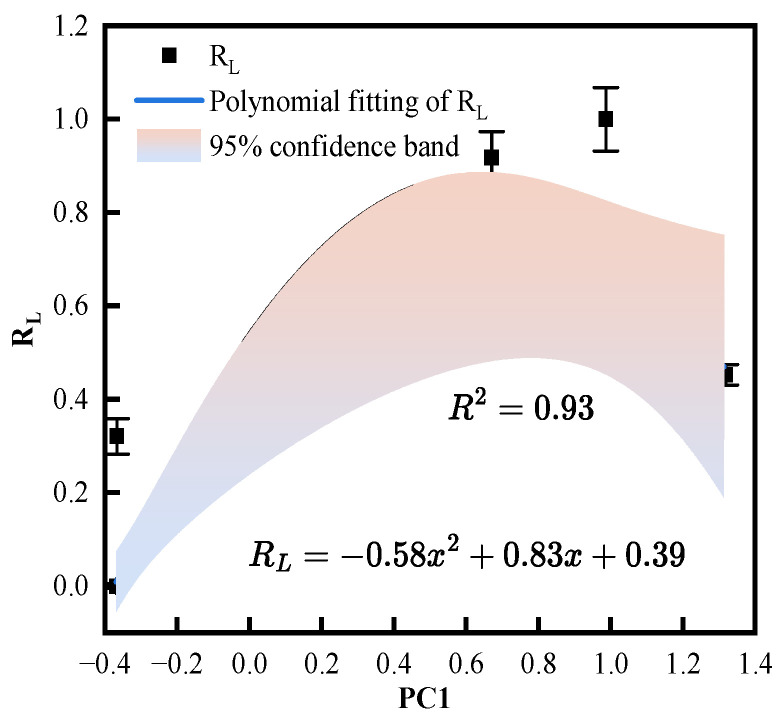
Polynomial fitting of principal component 1 (PC1) and *R*_L_.

Based on the primary component, a scatter plot of principal component 1 (PC1) against *R*_L_ is drawn, and a polynomial fit of the scatter produces the curve shown in Figure 14. The value of *R*_L_ is normalized from the range of [0, 1000] to [0, 1]. The resulting correlation coefficient of 0.93 indicates a good fit, meaning the fitting model accurately captures the trend and effectively describes the relationship between data points. An optimal range is identified within the principal component associated with an elevated *R*_L_. However, it is crucial to emphasize that the established relationship does not encompass road markings possessing extraordinarily high *R*_L_ values (>750 mcd·m^−2^·lx^−1^). Meanwhile, the model provides accurate predictions for the *R*_L_ of road markings with low-to-moderate retroreflective levels.

**Figure 15:**
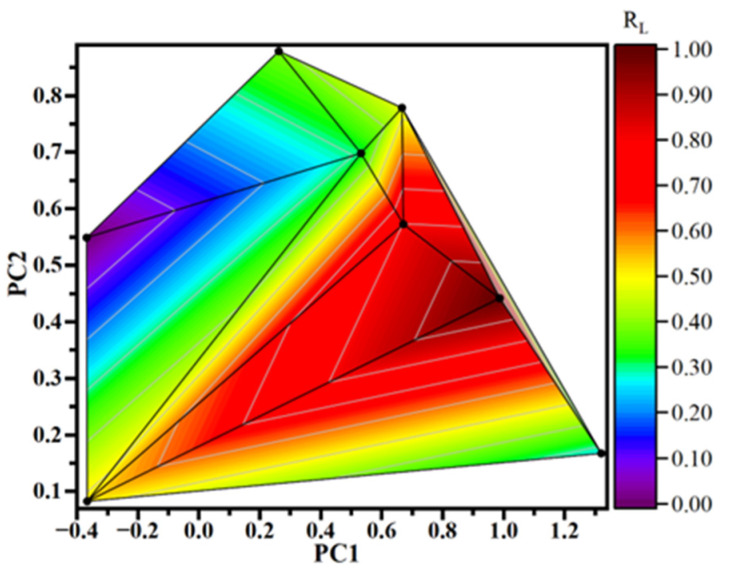
The GSMTA contour maps of PC1, PC2, and *R*_L_.

The contour map h of PC1, PC2, and *R*_L_ is shown in Figure 15. The cumulative percentage of variance accounted for is 91.70% when two principal components are chosen, with PC2 contributing 16.49%. Incorporating the second principal component significantly complicates the analysis of the impact of the evaluation indicators on the *R*_L_. Integrating the GSMTA graph with the contour map reveals that when PC1 values exceed 0.2, the corresponding normalized *R*_L_ values are greater than 0.2, indicating that the actual *R*_L_ exceeds 350 mcd·m^−2^·lx^−1^. This finding illustrates that road markings, when designed with a PC1 value greater than 0.2, can meet the necessary retroreflective performance standards in practical applications.

**Figure 16:**
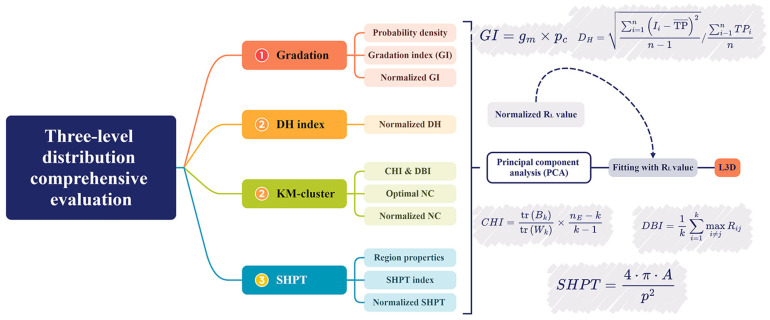
GSMTA of GB distribution.

Given the variability in *R*_L_ of road markings during actual construction, which is subject to a range of influencing factors, PC1 is chosen as the sole metric for assessing the distribution characteristics of GBs. This approach is designed to encapsulate the core impact of uniformity evaluation indicators on the *R*_L_ of traffic markings. The structure of the entire comprehensive evaluation system is illustrated in Figure 16.

The GSMTA index (L^3^D) is defined as follows:

(4) $$L^{3}D=0.564GI+0.452DH+0.401NC-0.580SHPT,$$

The relationship between *R*_L_ and L^3^D can be fitted as follows:

(5) $$R_{L}=-0.58L_{3}D^{2}+0.83L^{3}D+0.39$$

### 3.4. Field Application and Model Verification

To validate the performance of the GSMTA obtained in the laboratory, this study employs field data collected from test road sections. The reliability of the model is verified by comparing the measured values with the predicted values. The field application was carried out on Yan’an city (Shaanxi province, China), Nanchang city (Jiangxi province, China), and Taiyuan city (Shanxi province, China). The data was collected 1 month after the test sections were completed. At each test section, a randomized sampling protocol was implemented, with 50 measurement points selected for both *R*_L_ measurement and high-resolution image acquisition. To ensure data robustness, the recorded *R*_L_ values were statistically processed, with the arithmetic mean calculated as the representative metric for each sampling location. The image data of road markings collected from the expressway was processed based on the GSMTA framework.

**Figure 17:**
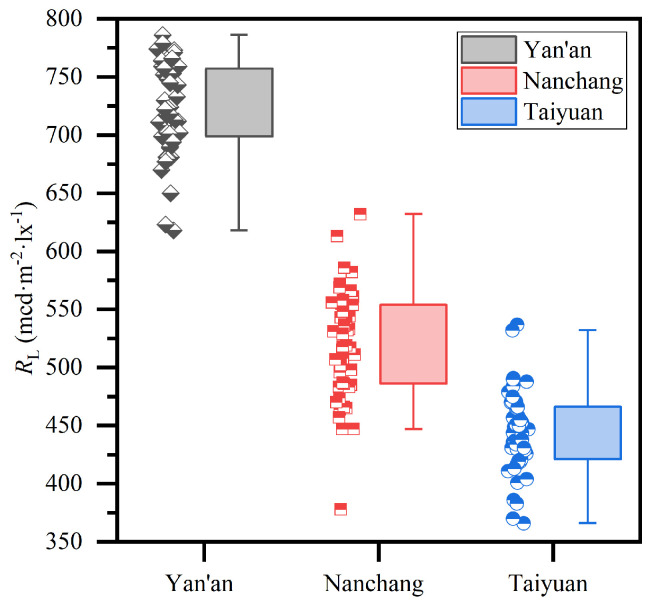
Measured values of *R*_L_ in Yan’an city, Nanchang city, and Taiyuan city.

Measured values of *R*_L_ in Yan’an city, Nanchang city, and Taiyuan city are shown in Figure 17. Analysis of the retroreflective coefficient distribution patterns across the experimental road sections revealed that MMA-based road markings, when combined with GBs exhibiting an RI of 1.7, achieved the highest initial retroreflective performance. In comparison, road sections utilizing thermoplastic coatings demonstrated significantly lower retroreflective coefficients than those with MMA-based coatings. Thermoplastic coatings exhibited 20–25% lower *R*_L_ than MMA systems. Notably, sections incorporating GBs with an RI of 1.5 exhibited markedly inferior initial retroreflective coefficients compared to all other tested sections.

As shown in Table 9, the 30%, 40%, 50%,60%, and 70% quantiles of the measured values were selected as the typical data to validate the GSMTA framework. The results indicate that the fitting relationship aligns with the actual state of road markings along the test section. The fitting results suggest that the predicted *R*_L_ closely matches the actual value when with the difference does not exceed 8% when the *R*_L_ is lower than 750 mcd·m^−2^·lx^−1^. However, when the actual *R*_L_ of the markings exceeds 750 mcd·m^−2^·lx^−1^, the discrepancy between predicted and actual values increases. This indicates that the fitting model accurately represents markings with *R*_L_ not exceeding 750 mcd·m^−2^·lx^−1^.

Although the relationship between principal components and *R*_L_ can be derived from the data mentioned above, the *R*_L_ of traffic markings is influenced by a multitude of factors, such as the embedding degree of GBs, the type of road marking coatings, the whiteness of the marking paint, and the degree of contamination and wear of the markings. It is impractical to accurately predict the *R*_L_ of traffic markings solely based on the uniform distribution characteristics of GBs. The derived formula can be utilized to assess the impact trend of the comprehensive uniformity indicator L^3^D on retroreflection and to evaluate the quality of GBs’ dispersion on traffic markings.

Furthermore, while the current YOLOv8-driven framework demonstrates high accuracy, its practical scalability across diverse geographic regions can be enhanced through transfer learning strategies. This would allow the model to adapt to localized pavement textures and paint formulations with minimal additional training data, ensuring the long-term viability of the GSMTA framework in routine infrastructure maintenance. Further research should consider factors including the type of marking paint, the RI of GBs, and the aging, wear, and contamination of markings, as well as the initial versus long-term service *R*_L_, to develop a comprehensive and accurate method for evaluating and predicting the performance of road markings.

## 4. Conclusions

This paper presents a novel GSMTA framework designed to assess the distribution state of GBs in road markings. Leveraging pixel-level segmentation of GBs facilitated by the YOLOv8-driven instance segmentation model, the following conclusions were drawn:

(1) The trained model significantly outperforms traditional methods (Otsu and Fast Random Forest) in GB segmentation, achieving a 15% higher accuracy and overcoming challenges from complex optical interferences (refraction, reflection, and shadows).
(2) The GSMTA framework synergistically evaluates granulometric properties, spatial heterogeneity, and morphological features of GBs through hierarchical indices. Field validation with three coating types confirmed its utility in evaluating initial retroreflective performance. MMA-based coatings with high-RI (1.7) GBs achieved optimal *R*_L_ (average being 721 mcd·m^−2^·lx^−1^). Thermoplastic coatings exhibited 20–25% lower *R*_L_ than MMA systems. Low-RI (1.5) GBs displayed notably degraded performance (average being 444 mcd·m^−2^·lx^−1^).
(3) The L^3^D metric exhibits high accuracy (errors ≤ 8%) for *R*_L_ prediction across validation sites within the *R*_L_ range below 750 mcd·m^−2^·lx^−1^ and demonstrates robustness at mid-range quantiles (30–70%), achieving average errors below ±5%
(4) While the L^3^D-*R*_L_ model excels for lower R_L_ values, it underestimates high *R*_L_ (>750 mcd·m^−2^·lx^−1^), highlighting the need for future optimization incorporating factors like coating chromaticity, RI of GBs, and paint type.

Despite the quantitative insights provided by this study, several limitations should be acknowledged for future research and practical deployment. Primarily because the deep learning model was trained on a restricted dataset comprising up to 150 macroscopic images, the validity of the current segmentation metrics and the resulting L^3^D evaluation system is intrinsically bound by the specific materials sampled in this study—namely, the evaluated glass bead gradations, binder formulations, and application practices. Consequently, extrapolating these quantitative findings to road markings with completely distinct regional construction practices or unfamiliar material types may introduce predictive uncertainties. Additionally, the current evaluation was conducted under idealized baseline conditions to systematically isolate the influence of GB gradation on retroreflective performance. In real-world engineering scenarios, factors such as surface wear, tire staining, and highly variable ambient lighting may pose additional challenges to segmentation robustness. Evaluating GB distribution solely as a function of *R*_L_ does not encompass the broader functional role of markings under real traffic conditions. To mitigate these environmental effects, the development of specialized acquisition hardware, featuring a standardized light-shielding enclosure and internal LED arrays, is envisioned to stabilize the optical environment for field inspections. Furthermore, to address the aforementioned regional limitations, while the current YOLOv8-driven framework demonstrates high accuracy within its specific training context, its practical scalability across diverse geographic regions can be enhanced through transfer learning strategies. This would allow the model to adapt to localized pavement textures and paint formulations with minimal additional training data, ensuring the long-term viability of the GSMTA framework in routine infrastructure maintenance. In addition, we identified the integration of daytime visibility (Qd), chromaticity degradation, and *R*_L_ trade-offs as the primary directive for future comprehensive modeling.

## Figures and Tables

**Table 1:** Gradation design schemes for both the single-size and mixed-gradation GBs.

Diameter Range (μm)	Percentage (%)		Diameter Range (μm)	Percentage (%)	
	Mix-L1	Mix-L2		Mix-S1	Mix-S2
1180~1000	25	20	600–850	30	30
1000~800	25	20	300–600	30	60
800~600	25	30	100–300	40	10
600~300	25	30	/	/	/

**Table 2:** Codes of test groups.

Code	SD6182	SD6812	SD6682	SD6362	Mix-S1	Mix-S2	Mix-L1	Mix-L2
Gradation/μm	1180–1000	1000–800	800–600	600–300	Mix-S1	Mix-S2	Mix-L1	Mix-L2

**Table 3:** The optimal results obtained by 30 rounds of hyperparameter evolution.

Hyperparameters	Datasets		
	30 Pictures	90 Pictures	150 Pictures
lr0	0.00942	0.01004	0.01074
lrf	0.00723	0.01002	0.00886
momentum	0.98	0.9209	0.82004
weight decay	0.00064	0.00052	0.00057
warmup epochs	3.11416	2.37788	3.2603
warmup momentum	0.75274	0.76152	0.77353
box	5.82296	6.79366	6.57075
cls	0.5314	0.47238	0.51159
dfl	1.34862	1.71744	1.23134
hsv_h	0.01613	0.01373	0.01968
hsv_s	0.52501	0.54535	0.75853
hsv_v	0.48736	0.40444	0.38552
translate	0.11645	0.07887	0.0923
scale	0.52574	0.5809	0.5
fliplr	0.59817	0.49828	0.49765
mosaic	0.9812	1	0.86039

Note: lr0/lrf: initial and final learning rate fractions; momentum: optimization momentum; weight_decay: L2 regularization penalty to mitigate overfitting; warmup_epochs/momentum: parameters for initial training stability; box/cls/dfl: objective loss weights for bounding box regression, classification, and distribution focal loss, respectively; hsv_h/s/v: color augmentation fractions (hue, saturation, and value); translate/scale/fliplr/mosaic: spatial data augmentation parameters representing image translation, scaling, left–right flip probability, and multi-image mosaic composition.

**Table 4:** The metrics corresponding to the optimal training rounds of different datasets.

Metrics	Datasets		
	30 Pictures	60 Pictures	90 Pictures
epoch	145	151	102
train/box_loss	0.33547	0.32796	0.31302
train/seg_loss	0.40738	0.35674	0.33899
train/cls_loss	0.30193	0.23291	0.22202
train/dfl_loss	0.72969	0.92243	0.6622
precision(B)	0.49236	0.99133	0.92108
recall(B)	0.48815	0.99389	0.97318
mAP50(B)	0.50312	0.99484	0.99473
mAP_50–95_(B)	0.43588	0.9259	0.9167
precision(M)	0.48146	0.98999	0.92406
recall(M)	0.47782	0.9893	0.97356
mAP50(M)	0.49434	0.99473	0.99468
mAP_50–95_(M)	0.35455	0.85122	0.87109
val/box_loss	0.4046	0.36133	0.33918
val/seg_loss	0.53672	0.39577	0.34119
val/cls_loss	0.3513	0.22793	0.22053
val/dfl_loss	0.74753	0.95037	0.68159
lr	0.00127	0.00125	0.00139

**Table 5:** Segmentation performance of different segmentation methods.

Segmentation Method	Algorithmic Characteristics	Visual Defects	Pixel Error
Otsu method	Global thresholding	Over-segmentation	+2% to 7%
Fast Random Forest	Local classification	Occasional shadow misidentification.	+1% to 5%
YOLOv8-seg	Deep contextual learning	Little, ignores optical artifacts	~1%

**Table 6:** GIs derived from image segmentation for both single-gradation and mixed-gradation GBs.

Type	SD6182	SD6812	SD6682	SD6362	Mix-S1	Mix-S2	Mix-L1	Mix-L2
GI	738.09	582.53	403.71	170.48	164.68	164.14	273.78	378.72

**Table 7:** Optimal NC of experimental groups with different gradations.

Type	SD6182	SD6812	SD6682	SD6362	Mix-S1	Mix-S2	Mix-L1	Mix-L2
NC	49	55	58	36	56	13	58	52

**Table 8:** The eigenvalues of the covariance matrix corresponding to the dataset **X**.

k	Eigenvalue	Variance Proportion	Cumulative Percent Variance	χ^2^	Degree of Freedom	Significance
1	0.3597	75.21%	75.21%	16.7581	9	0.0526
2	0.0789	16.49%	91.70%	6.0302	5	0.3033
3	0.0329	6.87%	98.57%	2.7125	2	0.2576
4	0.0068	1.43%	100.00%	0	0	0

**Table 9:** Comparison of measured data and predicted data of pavement markings.

Position	Type	Yan’an	Nanchang	Taiyuan
P30	*R*_L_-M *	701	497	430
	*R*_L_-P *	744	536	411
	error	6.13%	7.85%	−4.42%
P40	*R*_L_-M	712	511	435
	*R*_L_-P	735	482	409
	error	3.23%	−5.68%	−5.98%
P50	*R*_L_-M	722	519	445.5
	*R*_L_-P	672	533	467
	error	−6.93%	2.70%	4.83%
P60	*R*_L_-M	738	535	452
	*R*_L_-P	704	514	435
	error	−4.61%	−3.93%	−3.76%
P70	*R*_L_-M	753	545	462
	*R*_L_-P	680	517	486
	error	−9.69%	−5.14%	5.19%

*: *R*_L_-M denotes the actual value of *R*_L_, and *R*_L_-P denotes the predicted value of *R*_L_. P30 denotes the 30% quantile of the measured value.

## Data Availability

The original contributions presented in this study are included in the article. Further inquiries can be directed to the corresponding author.

## Abbreviations

The following abbreviations are used in this manuscript:

GBs	Glass beads
*R*_L_	Retroreflective luminance
LiDAR	Light Detection and Ranging
GSMTA	Granulometric–spatial–morphological triad assessment
MMA	Methyl methacrylate
RI	Refractive index
AP	Average precision
mAP	Mean average precision
IoU	Intersection-over-Union
GI	Gradation index
ROI	Region of interest
DH	Distribution homogeneity
NC	Number of clustering
CHI	Calinski–Harabasz index
DBI	Davies–Bouldin index
SHPT	Shape factor
PCA	Principal component analysis

## References

[1] Nance J., Sparks T.D. (2020). From streetlights to phosphors: A review on the visibility of roadway markings. Prog. Org. Coat..

[2] Lu X., Wu H., Li L., He R., Guo H. (2026). Confined photocatalysis and interfacial reconstruction for durability wettability in road markings. Colloids Surf. A Physicochem. Eng. Asp..

[3] Gui W.M., Liang L., Wang L., Zhang F. (2022). Cracking Resistance of Recycled Rubber Asphalt Binder Composed of Warm-Mix Additives. Materials.

[4] Li L., Lu X., Sun S., Zhang Y., He R. (2026). Enhancing interfacial trapping of mesoporous silica particles via grafting triterpenoid-saponin molecules to optimize the air-voids system of cement mortar under low atmospheric pressure. Constr. Build. Mater..

[5] Lyu L., Chen Y.X., Yu L.T., Li R., Zhang L., Pei J.Z. (2020). The Improvement of Moisture Resistance and Organic Compatibility of SrAl2O4: Eu2+, Dy3+ Persistent Phosphors Coated with Silica-Polymer Hybrid Shell. Materials.

[6] Smadi O., Hawkins N., Aldemir-Bektas B., Carlson P., Pike A., Davies C. (2014). Recommended Laboratory Test for Predicting the Initial Retroreflectivity of Pavement Markings from Glass Bead Quality. Transp. Res. Rec. J. Transp. Res. Board.

[7] He R., Shang Q., Lu X., Lu X., Guo W., Zhao J. (2026). Development of intelligent road markings: A review and outlook. J. Traffic Transp. Eng. (Engl. Ed.).

[8] Burghardt T.E., Maki E., Pashkevich A. (2021). Yellow thermoplastic road markings with high retroreflectivity: Demonstration study in Texas. Case Stud. Constr. Mater..

[9] Moghadam S.G., Pazokifard S., Mirabedini S.M. (2021). Silane treatment of drop-on glass-beads and their performance in two-component traffic paints. Prog. Org. Coat..

[10] Lee S., Koh E., Jeon S.-I., Kim R.E. (2024). Pavement marking construction quality inspection and night visibility estimation using computer vision. Case Stud. Constr. Mater..

[11] Soilán M., González-Aguilera D., del-Campo-Sánchez A., Hernández-López D., Del Pozo S. (2022). Road marking degradation analysis using 3D point cloud data acquired with a low-cost Mobile Mapping System. Autom. Constr..

[12] Nie Y.F., Wu W.J., Shan J.H., Peng H.X., Guo F.Y., Liu Y.H., Xiao J.J. (2026). Road Marking Distress Detection and Assessment Based on UAV Imagery. Materials.

[13] Feng X.W., Li B., Zhang Y., Xu Y.R. (2026). Study on Preparation of Long-Afterglow Luminescent Road-Marking Coatings and Simulation of Road Layout. Materials.

[14] Kulawik J., Kuczynski L. (2025). AI-Based Detection and Classification of Horizontal Road Markings in Digital Images Dedicated to Driver Assistance Systems. Appl. Sci..

[15] Nguyen S.D., Tran V., Tran T.S., Lee H.J., Flores J.M. (2023). Automated Segmentation and Deterioration Determination of Road Markings. J. Transp. Eng. Part B-Pavements.

[16] Babić D., Fiolić M., Babić D., Burghardt T.E. (2024). Systematic Testing of Road Markings’ Retroreflectivity to Increase Their Sustainability through Improvement of Properties: Croatia Case Study. Sustainability.

[17] Tardy H., Soilán M., Martín-Jiménez J.A., González-Aguilera D. (2023). Automatic Road Inventory Using a Low-Cost Mobile Mapping System and Based on a Semantic Segmentation Deep Learning Model. Remote Sens..

[18] Du Z.A., Yao Z.L., Wang S.S. (2023). Class-quantity and class-difficulty based methods for long-tailed road marking detection. J. Electron. Imaging.

[19] Li D.H., Liu T., Du P., Ma T.E., Liu S.T. (2025). Context-aware and boundary-optimized model for road marking instance segmentation using MLS point cloud intensity images. Int. J. Digit. Earth.

[20] Lu X., Shang Q., He R., Yao W., Guo H. (2024). Visible characteristics and durability assessment of methyl methacrylate-based luminescent road marking using unencapsulated SrAl_2_O_4_: Eu^2+^, Dy^3+^ under coupling service conditions. Constr. Build. Mater..

[21] Sruthy S., Prakash A.J. (2025). DeepVisionMark: Enhancing Autonomous Navigation Through Road Mark Detection Using Advanced Deep Learning Model. Int. J. Intell. Transp. Syst. Res..

[22] Chen S.Y., Zhang Z.X., Zhong R.F., Zhang L.Q., Ma H., Liu L.R. (2021). A Dense Feature Pyramid Network-Based Deep Learning Model for Road Marking Instance Segmentation Using MLS Point Clouds. IEEE Trans. Geosci. Remote Sens..

[23] Fan J., Li Z.L., Wang Y. (2024). Enhanced Road Marking Point Cloud Extraction Method Using Improved RandLA-Net. Laser Optoelectron. Prog..

[24] Guo C., Su Y.H., Zuo C. A Heterogeneous Semi-supervised Learning Network for Road Damage Detection. Proceedings of the 2024 9th International Conference on Intelligent Computing and Signal Processing, ICSP.

[25] Manahiloh K.N., Abera K.A., Motalleb Nejad M. (2018). A Refined Global Segmentation of X-Ray CT Images for Multi-phase Geomaterials. J. Nondestruct. Eval..

[26] Wei Z., Zhang X., Ma L., Zhang Y., He R. (2026). Early-age pore-structure evolution in internally cured concrete with super-absorbent polymers: A multiscale study. Constr. Build. Mater..

[27] Lagahit M.L.R., Liu X., Xiu H.Y., Kim T., Kim K.S., Matsuoka M. (2024). Learnable Resized and Laplacian-Filtered U-Net: Better Road Marking Extraction and Classification on Sparse-Point-Cloud-Derived Imagery. Remote Sens..

[28] Chen C., Jia Y.D., Yu L.L., Liu T. (2026). A Multi-Scale Context Fusion Network With Boundary Refinement for Road Marking Segmentation. IEEE Access.

[29] Li H., Gao K., Liang H., Zhu H., Yang Z., Wang Q. (2024). An efficient out-of-distribution pixel-level crack detection framework using prior knowledge. J. Build. Eng..

[30] Chen S., Li Y., Zhang Y., Yang Y., Zhang X. (2024). Soft X-ray image recognition and classification of maize seed cracks based on image enhancement and optimized YOLOv8 model. Comput. Electron. Agric..

[31] Jocher G., Chaurasia A., Qiu J. (2023). *Ultralytics YOLO*, version 8.0.197. https://github.com/ultralytics/ultralytics.

[32] Du J., Ma L.F., Li J., Qin N.N., Zelek J., Guan H.Y., Li J.A.T. (2024). RdmkNet & Toronto-Rdmk: Large-Scale Datasets for Road Marking Classification and Segmentation. IEEE Trans. Intell. Transp. Syst..

[33] Iparraguirre O., Iturbe-Olleta N., Brazalez A., Borro D. (2022). Road Marking Damage Detection Based on Deep Learning for Infrastructure Evaluation in Emerging Autonomous Driving. IEEE Trans. Intell. Transp. Syst..

[34] Wu J.J., Liu W., Maruyama Y. (2024). Street View Image-Based Road Marking Inspection System Using Computer Vision and Deep Learning Techniques. Sensors.

[35] Torralba A., Russell B.C., Yuen J. (2010). LabelMe: Online Image Annotation and Applications. Proc. IEEE.

[36] Chen T., Dai J., Dong B., Zhang T., Xu W., Wang Z. (2024). Road marking defect detection based on CFG_SI_YOLO network. Digit. Signal Process..

[37] Sheng S., Formosa N., Feng Y., Quddus M. (2026). Clip-based road-marking detection with LLM-guided driving prompts. Int. J. Appl. Earth Obs. Geoinf..

[38] Liu J., Yuan L., Wang Z., Jing H., Shao T., Chen H. (2023). A new method for evaluating the uniformity of steel slag distribution in steel slag asphalt mixture based on deep learning. Constr. Build. Mater..

[39] Syarofina S., Bustamam A., Yanuar A., Sarwinda D., Al-Ash H.S., Hayat A. (2021). The distance function approach on the MiniBatchKMeans algorithm for the DPP-4 inhibitors on the discovery of type 2 diabetes drugs. Procedia Comput. Sci..

[40] Gonzalez K., Misra S. (2022). Unsupervised learning monitors the carbon-dioxide plume in the subsurface carbon storage reservoir. Expert Syst. Appl..

[41] Ros F., Riad R., Guillaume S. (2023). PDBI: A partitioning Davies-Bouldin index for clustering evaluation. Neurocomputing.

[42] van der Walt S., Schonberger J.L., Nunez-Iglesias J., Boulogne F., Warner J.D., Yager N., Gouillart E., Yu T. (2014). scikit-image: Image processing in Python. PeerJ.

[43] Kaviani-Hamedani F., Esmailzade M., Adineh K., Shafiei M., Shirkavand D. (2023). Quantifying three-dimensional sphericity indices of irregular fine particles from 2D images through sequential sieving tests. Granul. Matter.

[44] Yuan J., Masuko S., Shimazaki Y., Chai J. (2022). Researching the design of a glass-bead retro-reflective material to reduce downward reflection for urban heat island mitigation. Mater. Today Sustain..

